# Lebensqualität von Patienten mit hydraulischen penilen Implantaten bei erektiler Dysfunktion in Bezug auf die sexuelle Zufriedenheit des Patienten und seinem/seiner Partner/Partnerin

**DOI:** 10.1007/s00120-020-01418-z

**Published:** 2020-12-23

**Authors:** K.-M. Arndt, A. Chomicz, K.-P. Jünemann, D. Osmonov

**Affiliations:** 1 Klinik für Urologie und Kinderurologie, Universitätsklinikum Schleswig-Holstein Campus Kiel, Kiel, Deutschland; 2 Professor-Plücker-Str. 5, 38302 Wolfenbüttel, Deutschland

**Keywords:** Penisprothesenimplantation, Erhebungen und Fragebögen, Infektionen, PDE-5-Hemmer, Autoinjektion, Penile prosthesis, Surveys and Questionnaires, Infections, PDE‑5 inhibitors, Autoinjection

## Abstract

**Hintergrund:**

Die erektile Dysfunktion ist eine Erkrankung mit einer stetig wachsenden Prävalenz in der männlichen Bevölkerung. Die Penisprothesenimplantation gilt als effektive Therapieoption.

**Fragestellung:**

Ziel der durchgeführten Umfrage war daher, die sexuelle Zufriedenheit und somit die Lebensqualität von Patienten mit einer Penisprothese zu untersuchen und zusätzlich die Lebensqualität und sexuelle Zufriedenheit der Partner zu ermitteln.

**Material und Methoden:**

Die Studie wurde ausschließlich mit Patienten, welche am Universitätsklinikum Schleswig-Holstein (UKSH), Campus Kiel operiert wurden, durchgeführt. Die zur Verfügung stehende untersuchte Fallzahl an Patienten betrug 121. Für die seltene Indikation der PPI („penis prosthesis implant“) ist dies eine beachtliche Fallzahl. Hierfür wurden die Teilnehmer im Alter 44–80 Jahren und ihre jeweiligen Partner mittels validierten (EDITS [„erectile dysfunction inventory of treatment satisfaction“], EDITS Partner) Fragebögen befragt. Das mittlere Alter der befragten Patienten betrug 63 Jahre. Von 121 versendeten Fragebögen wurden 46 von den männlichen Patienten beantwortet. Daraus ergibt sich eine Antwortrate von 38,0 %.

**Ergebnisse:**

Als Hauptergebnis konnte nachgewiesen werden, dass die Zufriedenheitsrate in den meisten Fällen mit einem hohen Ergebnis und somit einer hohen Zufriedenheit der Patienten (80,97 % mittlerer EDITS) sowie deren Partner (65 %) einhergeht.

**Schlussfolgerung:**

Die Penisprothesenimplantation liefert in Bezug auf die allgemeine sexuelle Zufriedenheit exzellente Ergebnisse und führt zu einer hohen Lebensqualität für Patienten und Partner.

## Abstract (en)

**Background:**

Erectile dysfunction is a condition that shows a continuously growing prevalence in the male population. The penis prosthesis implant (PPI) qualifies as an effective form of therapy.

**Objectives:**

The aim of this study was to analyze the sexual satisfaction rate and quality of life in patients who had suffered from erectile dysfunction and who were treated with a penile prosthesis. The patient’s partners were also surveyed.

**Methods:**

We collected data from patients who underwent surgery in the Center of Excellence for Penile Implants, University Hospital Schleswig–Holstein, Campus Kiel, Kiel, Germany. Questionnaires with validated scores (EDITS, EDITS Partner) were sent to all patients and their partners via mail.

**Results:**

The satisfaction rate in this study was high which shows that the patients and partners are pleased, and the high sexual satisfaction rate led to a higher quality of life.

**Conclusion:**

The penile prosthesis implantation as a last option of therapy for erectile dysfunction is useful and brings more than adequate results.

## Einleitung

### Erektile Dysfunktion

Die erektile Dysfunktion (ED) beeinflusst die Lebensqualität (LQ) der Betroffenen und deren Partner negativ [1]. Weltweit gibt es weit über 150 Mio. Erkrankte und die Prävalenz steigt. In der „The Massachusetts Male Aging Study“, welche zwischen 1987 und 1989 durchgeführt wurde, litten 52 % aller Befragten und sonst gesunden 40- bis 70-jährigen Männer an milder, moderater oder ausgeprägter ED [2]. Im „Cologne Male Survey“ von Braun et al. Betrug die Prävalenz bei 8000 befragten Männern zwischen 30 und 80 Jahren 19,2 % [3].

Die Therapieoptionen umfassen ein weites Feld an Möglichkeiten, da die Ätiologie der ED vielfältig ist. Seit Erstbeschreibung des hydraulischen Penisimplantats, der „Scott-Bradley-Timm-Prosthesis“ im Jahre 1973, wird diese Methode gewählt, wenn konservative Therapiemethoden nicht erfolgreich sind oder bei therapierefraktären Patienten [4]. Hydraulische Modelle sind heutzutage am weitesten verbreitet, da diese kosmetisch und physiologisch der ursprünglichen Anatomie des Penis am nächsten kommen. Die Zufriedenheit des Patienten und die Akzeptanz sowie die Zufriedenheit der Partner mit dem System ist mehreren Studien zufolge nach sehr hoch [5, 6].

Infektionen sind die meist gefürchtetsten Komplikationen bei der Implantation von Penisprothesen. In der Studie von Cosentino et al. [7] wird beschrieben, dass die Implantation von Penisprothesen (PP) effektiv und sicher ist. Es ist jedoch äußerst wichtig, die Infektionsrate so gering wie möglich zu halten. Hierzu sollten die Chirurgen strikten Hygienemaßnahmen folgen.

## Material/Methoden

### Patientenkollektiv

Die Patienten wurden zwischen Januar 2012–März 2017 in der Klinik für Urologie und Kinderurologie des Universitätsklinikum Schleswig-Holstein Campus Kiel (UKSH Kiel) operiert. Die Durchführung der Studie brachte es mit sich, dass die zur Verfügung stehenden Patienten und deren Bereitschaft nur eine kleine Fallzahl (für diese seltene Indikation der PPI aber dennoch beachtliche Patientenzahl) erwarten ließ. Von 121 versendeten Fragebögen wurden 46 von den männlichen Patienten und ihren Partnern beantwortet. 75 (62,0 %) der Befragten haben nicht an der Umfrage teilgenommen. Daraus ergibt sich eine Antwortrate von 38,0 %.

In der Studie mit dem Ethikvotumaktenzeichen 452/20 wurde untersucht, inwieweit die Patienten und Partner den Einfluss der Operation und des Penisimplantats auf ihre Lebensqualität in Bezug auf die sexuelle Zufriedenheit retrospektiv beurteilen.

### Fragebogen/Zielsetzung

Für diese retrospektive Studie wurde ein Fragebogen mit validierten Scores für die Patienten und ihre Partner in Kiel erstellt, welcher sich mit dem Grad der Zufriedenheit (ZR), ermessen anhand unterschiedlicher Fragen, befasst. Dieser wurde auf postalischem Weg an die Patienten und ihre Partner verschickt. Im ersten Abschnitt des Fragebogens wurden allgemeine Daten der Patienten aufgenommen. Diese Studie befasste sich hauptsächlich mit dem Teil des Fragebogens, welcher sich auf die Zufriedenheit des Patienten bezieht. In einigen Auswertungen wurde auf verschiedene Faktoren des allgemeinen Fragebogenteils eingegangen. Der „erectile dysfunction inventory of treatment satisfaction“ (EDITS) sowie der „erectile dysfunction inventory of treatment satisfatction partner survey“ (EDITS Partner) waren inbegriffen [8].

Der EDITS und der EDITS Partner befassen sich v. a. mit Fragen bezüglich des Sexuallebens des Patienten und des jeweiligen Partners [8]. Die Studienergebnisse sollen dazu dienen, Patienten, welche an einer ED leiden und deren Partner ein möglichst zufriedenstellendes Sexualleben nach Implantation einer Penisprothese zu ermöglichen. Die ZR wird anhand verschiedener Faktoren beurteilt: die allgemeine Zufriedenheitsrate (ZR), erektile Funktion/Aktivität, Nutzen prä- und postoperativ und das kosmetische Aussehen des Penis im erigierten und schlaffen Zustand. Ferner wird die Natürlichkeit der sexuellen Interaktion betrachtet.

Die Daten des Fragebogens wurden anhand verschiedener statistischer Tests analysiert und ausgewertet. Mit Hilfe von Kreuztabellen wurde die Häufigkeit in absoluter und relativer Häufigkeit dargestellt. Die Unabhängigkeit wurde mittels χ^2^-Test getestet. Zusätzlich wurde der t‑Test für zwei abhängige Stichproben verwendet, um zu testen, ob die Mittelwerte der Stichproben verschieden sind. In Tab. 4 sind die Vorerkrankungen der Patienten beschrieben. Hierbei ist zu erwähnen, dass einige Patienten mehrere Erkrankungen haben, so dass sich letztlich eine Prozentzahl >100 % ergibt. Zu den Vorerkrankungen gehören Schwellkörperfibrose, Penisdeviation, Zustand nach radikaler retropubischer Prostatektomie, Diabetes mellitus Typ 1 und Typ 2, koronare Herzerkrankungen, arterieller Hypertonus, Depression, neurologische Erkrankungen und Hypercholesterinämie (vgl. Tab. 4).

## Ergebnisse

### Patientenzufriedenheit

#### Allgemeine Zufriedenheit und Zufriedenheit mit dem Implantat

Der Altersmittelwert der Teilnehmer beträgt 63 Jahre. Hierbei wird deutlich, dass die ED mit steigendem Alter zunimmt. Die Patienten wurden mit PP der Marke Coloplast Titan PP® (Coloplast, Hamburg) versorgt (Tab. 1).

**Table 1:** 

Implantat	Häufigkeit beantwortet absolut	Häufigkeit beantwortet relativ (%)
Coloplast Titan PP®	44	95,65
Gesamt	46	100

**Figure 1:**
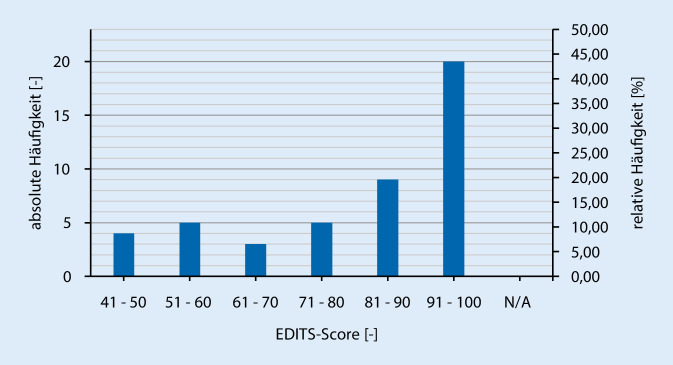

Die allgemeine ZR (als Summe verschiedener Fragen) wurde durch die Auswertungen des Fragebogens mittels Fragen des EDITS ausgedrückt. Zur Berechnung des EDITS-Scores wurde der Mittelwert aller beantworteten Fragen herangezogen und mit dem Faktor 25 multipliziert. Hierdurch ergaben sich EDITS-Scores zwischen 0 und 100, wobei ein höherer EDITS-Score auf eine höhere allgemeine Zufriedenheit hinweist (Abb. 1). Insgesamt kann aufgrund des hohen Mittelwertes und der Tatsache, dass 63,0 % der Befragten einen höheren EDITS-Score als 80 erzielten bzw. nur 19,6 % einen geringeren Score als 61 aufwiesen, davon ausgegangen werden, dass die allgemeine Zufriedenheit der Patienten hoch ist und damit einhergehend die meisten der befragten Patienten mit der PP zufrieden sind. Nach der Frage der Zufriedenheit mit dem Implantat allgemein, gaben (80,4 %) befragen Patienten den Punktwert 3 (etwas zufrieden) oder 4 (sehr zufrieden) an. Dies deutet auf eine allgemein gute Zufriedenheit hin.

#### Einfluss der PP auf das Sexualleben (post-/präoperative sexuelle Zufriedenheit/Aktivität/kosmetisches Aussehen, Weiterempfehlung/Wiederholung)

Die explizite Frage nach der sexuellen Zufriedenheit nach der Operation im Vergleich zur präoperativen sexuellen Zufriedenheit ergab, dass 58,7 % der Patienten eine erhöhte Zufriedenheit, 21,7 % neutral/keine Veränderungen und 17,4 % eine geringere Zufriedenheit angaben. Des Weiteren betrachteten wir die sexuelle Aktivität nach PPI. Hier zeigte sich, dass der größte Anteil der Patienten (43,5 %) ihre postoperative sexuelle Aktivität als sehr gut und 45,7 % der Patienten als befriedigen empfinden. Nur 4,4 % der Patienten bewerteten die postoperative Aktivität als schlecht.

**Figure 2:**
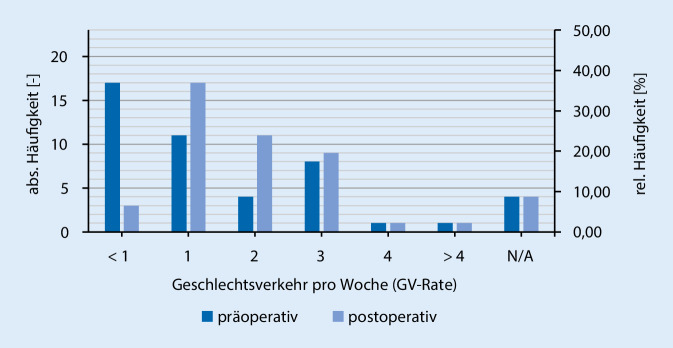

**Figure 3:**
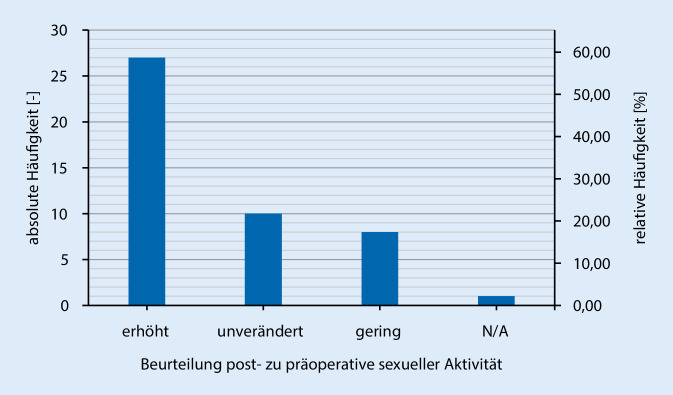

**Figure 4:**
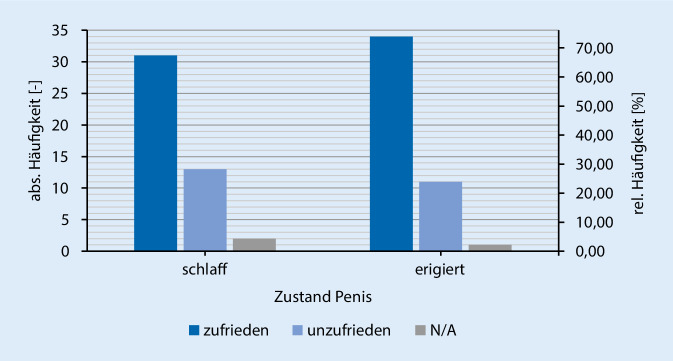

Des Weiteren zeigte sich eine Zunahme der Geschlechtsverkehrsrate postoperativ zu präoperativ (Abb. 2), sowie auch ein Anstieg der sexuellen Aktivität (Abb. 3). Hierbei zeigt sich, dass >65 % der Patienten mit dem kosmetischen Aussehen des Penis sowohl im schlaffen als auch erigiertem Zustand zufrieden sind (Abb. 4). Der größte Teil (84,8 %) der Patienten würde sich zudem sehr wahrscheinlich erneut für eine Implantation der PP entschließen und die Therapieoption auch anderen Männern empfehlen.

### Partnerzufriedenheit

**Figure 5:**
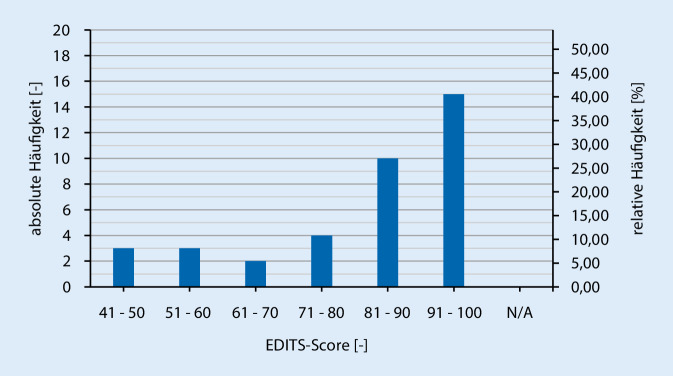

**Figure 6:**
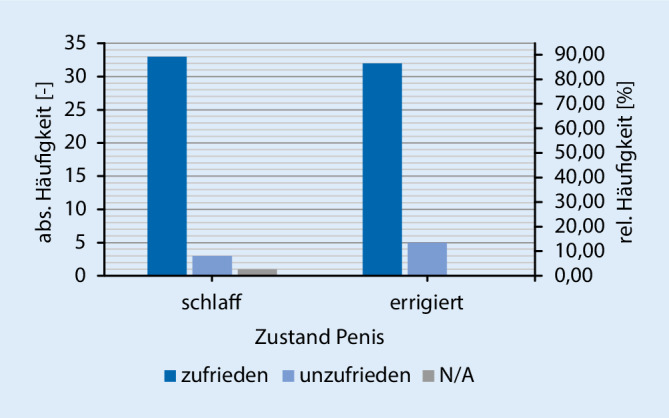

Zudem wurde die Frage nach der Partnerzufriedenheit separat betrachtet. Mit einer Antwortrate von 26,45 % konnten die Ergebnisse der Partner ausgewertet werden. Hierbei konnte festgestellt werden, dass die Partner i. Allg. eine hohe Zufriedenheitsrate angaben (Mittelwert des EDITS Partners 84,7 %, Abb. 5). Die Partner geben demnach zusätzlich eine erhöhte sexuelle Zufriedenheit nach Implantation an (46,8 %). Hierbei wird deutlich, dass die postoperative sexuelle Zufriedenheit im Vergleich zu der präoperativen Zufriedenheit deutlich gesteigert ist. Vergleichend kann darüber hinaus gesagt werden, dass die Patienten und Partner gleichermaßen mit dem kosmetischen Aussehen des Penis nach der Operation zufrieden sind (Abb. 6).

## Diskussion

Die ED stellt einen maßgeblichen Grund für eine erhebliche Einschränkung der LQ dar. Die Implantation einer Penisprothese repräsentiert die einzige Therapieoption für Patienten mit therapierefraktärer ED, Kontraindikationen zu konservativen Methoden oder unzureichendem Ergebnis [9]. Kontraindikationen der PDE-5-Hemmer (Phosphodiesterase) umfassen u. a.: Hypogonadismus, und Überempfindlichkeit, kardialen Vorerkrankungen sowie die gleichzeitige Einnahme von Nitraten, da diese zu einer lebensbedrohlicher Hypotension führen kann. Darüber hinaus können Vakuumpumpen, Schwellkörperautoinjektionstherapien und die intraurethrale Applikation von Prostaglandin E1 bei falschem Gebrauch zu Verletzungen führen [10].

Die ZR und somit auch die LQ der Patienten ist seit der ersten Beschreibung einer hydraulischen Penisprothese durch Scott et al. [4] deutlich zunehmend. Laut einer Studie von Bernal und Henry [11] waren die Patienten nach PPI i. Allg. mit dem Implantat und der sexuellen Funktion dieser, trotz ihrer statistischen Variabilität und Mangel an validierten Fragebögen, zufrieden. Hierzu betrachtete diese Studie die Literatur von der Zufriedenheit von Patienten nach PPI der letzten 20 Jahre.

Die 9 inkludierten Studien, welche validierte Fragebögen nutzen, zeigten eine hohe ZR. Ein zusätzlich wichtiger Aspekt der allgemeinen und v. a. der sexuellen Zufriedenheit der Patienten ist die Zufriedenheit des Partners. Eine Studie zu diesem Thema wurde von Porena et al. [12] beschrieben. Hier wurde die Wirkung der PP auf die sexuelle Zufriedenheit von Paaren betrachtet. 82 % der Patienten und ihrer Partner waren ein Jahr nach der Operation zufrieden. Als Hauptergebnis konnte nachgewiesen werden, dass die ZR in den meisten Fällen mit einem hohen Ergebnis und somit einer hohen ZR der Patienten, sowie deren Partner einhergeht.

In einer Studie aus dem Jahr 2013 zeigten sich ähnliche Zahlen. In dieser Studie konnten 65 Patientenergebnisse inkludiert werden. Das mittlere Alter der Patienten betrug 56 Jahre. Hierbei wurde die ZR anhand zweier validierter Scores nach AMS 700CX/CXR®-Prothesenimplantation (Boston Scientific, Boston, MA, USA) analysiert. Die Ergebnisse zeigten eine hohe ZR (Mittelwert EDITS 73,11) und somit schlussfolgernd eine hohe LQ, auch in Bezug auf die sexuelle Zufriedenheit [13]. In der Studie von Casabe et al. [6] lag die ZR bei Werten um >75,6 %. 60 Patienten mit einem mittleren Alter von 61,7 Jahren wurden untersucht. Davon erhielten 36 Patienten eine Penisprothese der Marke Spectra® (Boston Scientific) und 24 Patienten Genesis® (Coloplast). Eine weitere Studie aus dem Jahr 2015 erzielte eine Rate von 86,8 % in der gesamten ZR, sowie ein 81,1 %iges Ergebnis zugunsten der Steigerung des Sexuallebens. 53 der 74 Befragten haben den Fragebogen beantwortet. Das mittlere Lebensalter betrug 57 Jahre. Alle Patienten erhielten ein Implantat der Firma AMS 700 CXM® [5].

In der Studie von Rajpurkar et al. [14] wurden drei Therapieoptionen (orale PDE-5-Hemmer, intrakavernöse Injektion und die PP) verglichen. Hierbei zeigte sich eine höhrere ZR bei den Patienten mit PP im Vergleich zu den anderen Therapieoptionen. Durch die verschiedenen statistischen Auswertungen konnte bestätigt werden, dass es eine allgemein hohe ZR (76,2 %) nach PPI gibt. Dies wird auch bei der Partnerbefragung deutlich. Hierbei hatten 65 % der Partner einen EDITS-Score über 81 erzielt, was einer hohen Zufriedenheit entspricht. Die sexuelle Aktivität nach der Implantation wurde von 47,6 % der Patienten als sehr gut und von 35,7 % befriedigend bewertet. 46,8 % der Partner bestätigten zudem, dass die sexuelle Aktivität nach Implantation gesteigert war. Hieraus lässt sich zusätzlich schließen, dass die Implantation zu guten Ergebnissen und einer positiven Auswirkung auf die LQ für die Patienten und Partner führt. Die Befragung der Partner ist demnach essentiell wichtig für die Beurteilung der ZR.

In der Studie von Gittens et al. [15] wird deutlich, dass ein gewisses Risiko für eine sexuelle Dysfunktion der Partner besteht, sobald die implantierten Patienten eine niedrige ZR angeben. Das kosmetische Aussehen des Penis im schlaffen und erigiertem Zustand zeigte, dass die Mehrzahl der Patienten (>65 %) zufrieden sind. Des Weiteren würden 84,8 % der Patienten die PPI anderen Männern mit einer sehr hohen Wahrscheinlichkeit weiterempfehlen. Ähnlich hohe Weiterempfehlungsraten (75 % und 72 %) zeigte die Studie von Brinkmann et al. [16]. Die insgesamt 190 positiven Rückläufer wurden mit Prothesen der Marken AMS 700 Series®, Mentor Alpha 1® und Mentor Alpha NB® (Mentor Corp., Santa Barbara, CA, USA) therapiert.

Wir haben somit festgestellt, dass sowohl die allgemeine Zufriedenheit, als auch die sexuelle Zufriedenheit der Patienten und ihrer Partner nach einer Penisprothesenimplantation mit einer hohen Rate einhergeht und es zudem eine große Weiterempfehlungsrate dieser Therapieoption zeigt. Des Weiteren konnten wir feststellen, dass das kosmetische postoperative Ergebnis auch mit einer hohen Zufriedenheit einhergeht. Schlussfolgernd kann gesagt werden, dass die Penisprothesenimplantation ein exzellentes Ergebnis hinsichtlich der allgemeinen Zufriedenheit, sexuellen Zufriedenheit und somit Lebensqualität der Patienten und deren Partner liefert (Tab. 2 und 3).

**Table 2:** 

Autor/Publikation	Patienten (*n*)	Partner (*n*)	Zufriedenheit (%) Patient/Partner
Porena et al. [12]	46	/	82
Montorsi et al. [17]	185	120	92/96
Carvalheira et al. [18]	47	–	79
K.-M. Arndt et al. (exp. 2020)	46	37	81/84 (mittlerer EDITS)

*EDITS* „erectile dysfunction inventory of treatment satisfaction“

**Table 3:** 

Allgemeine ZR	Implantat ZR	Sexuelle ZR nach Operation	Weiterempfehlung	Wiederholung	Kosmetisches Aussehen
80,97 Mittelwert (%, EDITS)	80,4	>85	84,8	84,8	>65

*EDITS* „erectile dysfunction inventory of treatment satisfaction“, *ZR* Zufriedenheit

**Table 4:** 

Vorerkrankungen	Absoluter Wert (*n*)	Relativer Wert (%)
Schwellkörperfibrose	5	10,87
Radikale retropubische Prostatektomie (Zustand nach RRP)	6	13,04
Penisdeviation	3	6,52
Diabetes Mellitus Typ 1	2	4,35
Diabetes Mellitus Typ 2	10	21,74
Arterieller Hypertonus	23	50,00
Koronare Herzkrankheit	6	13,04
Hypercholesterinämie	8	17,39
Depression	5	10,87
Neurologische Erkrankungen	3	6,52
Keine Vorerkrankungen	13	28,26

## Fazit für die Praxis

- Die Penisprothesenimplantation (PPI) liefert in Bezug auf die allgemeine sexuelle Zufriedenheit exzellente Ergebnisse.
- Sie zeigt eine deutliche Verbesserung des Sexuallebens der Patienten und Partner.
- Schlussfolgernd ergibt sich eine deutliche Verbesserung der Lebensqualität.
- Die PPI ist Therapieoption bei refraktärer und/oder kontraindizierter vorheriger konservativer Therapie.
- Die Aufklärung der Patienten und Partner ist notwendig.
- Weiterempfehlungs- und Wiederholungsraten sind hoch.
